# „iFightDepression“ im stationären Setting: Evaluation eines internetbasierten, therapeutenbegleiteten Selbstmanagementprogramms auf einer Spezialstation für affektive Störungen

**DOI:** 10.1007/s00115-021-01214-w

**Published:** 2021-10-15

**Authors:** Julian Schwarz, Nicole Mauche, Caroline Oehler, Christine Rummel-Kluge, Ulrich Hegerl, Maria Strauß

**Affiliations:** 1 Department für Psychische Gesundheit, Klinik und Poliklinik für Psychiatrie und Psychotherapie, Universitätsklinikum Leipzig, AöR, Semmelweisstr. 10, 04103 Leipzig, Deutschland; 2 Stiftung Deutsche Depressionshilfe, Leipzig, Deutschland; 3 Senckenberg Professur, Klinik für Psychiatrie, Psychosomatik und Psychotherapie, Goethe-Universität Frankfurt am Main, Frankfurt am Main, Deutschland

**Keywords:** E‑Mental-Health, Depression, IKVT, IFightDepression, Selbstmanagement, E‑mental health, Depression, Cognitive behavioral therapy, Self-management, Clinical study

## Abstract

**Hintergrund:**

E‑Mental-Health (EMH) spielt im ambulanten Versorgungssetting depressiver Störungen zunehmend eine Rolle. Ziel dieser Studie war die Implementierung und Evaluierung der Anwendbarkeit und des Nutzens des onlinebasierten Selbstmanagementprogramms „iFightDepression“ (iFD) als Zusatzangebot im Rahmen einer leitliniengerechten Behandlung auf einer Spezialstation für affektive Störungen.

**Methodik:**

Es wurden insgesamt 78 stationäre PatientInnen mit einer unipolaren Depression unterschiedlichen Schweregrades (ICD-10 F32.0‑3, F33.0-3) oder einer Dysthymie (F34) rekrutiert. Die Interventionsdauer mit dem iFD-Tool belief sich vom Zeitpunkt der stationären Aufnahme bis zur Entlassung und wurde vom Stationspersonal begleitet. Die Erhebung der Symptomschwere sowie von Parametern zur Behandlungserwartung und Therapievorerfahrung erfolgte online vor der Intervention (T0), die Interventionszufriedenheit wurde unmittelbar vor der stationären Entlassung (T1) mittels eines Paper-pencil-Fragebogens erfasst.

**Ergebnisse:**

Von den 78 Teilnehmenden loggten sich 42 mindestens einmal in das iFD-Tool ein. Es zeigten sich moderat hohe Erwartungswerte sowie leicht überdurchschnittliche Zufriedenheitswerte bezüglich der Behandlung. 67 % der aktiven NutzerInnen gaben an, das iFD-Tool poststationär weiter nutzen zu wollen. Wesentliche Gründe gegen die Nutzung waren eine kurze Aufenthaltsdauer, eine schwere depressive Symptomatik und fehlende Medienkompetenz.

**Diskussion:**

Eine Implementierung des iFD-Tools im stationären Setting ist prinzipiell machbar und führte zu positiven Rückmeldungen seitens der NutzerInnen. Auch konnten sich die meisten aktiv an der Studie Teilnehmenden eine weitere Nutzung des iFD-Tools nach der Entlassung gut vorstellen. Allerdings weisen die insgesamt geringen Nutzungs- (42/78, 54 %) und Komplettierungsraten auf Nutzungsbarrieren hin, deren Überwindung entsprechende Anpassungen der Intervention speziell für den stationären Einsatz erforderlich machen.

## Abstract (en)

**Background:**

E‑mental health mainly plays a role in the outpatient treatment of patients with depressive disorders. The goal of this study was to implement and evaluate the web-based, therapist-guided self-management tool “iFightDepression” (iFD) to clarify if there is a benefit for inpatient use.

**Material and methods:**

In this study 78 inpatients with affective disorders (ICD-10 F32.0‑3, F33.0-3) or dysthymia (F34) were recruited. The intervention duration with the iFD tool went from admission until discharge, therapeutic support was granted by the ward staff. Symptom severity, intervention expectations and experience with therapy were processed in an online questionnaire before the intervention (T0) while intervention satisfaction was captured after the intervention shortly before discharge (T1) in a paper-pencil questionnaire.

**Results:**

Out of 78 participating inpatients 42 used the iFD tool at least once. Moderate to high levels of expectation regarding the iFD tool and mildly above-average level of satisfaction after the intervention were observed. Of the active users 67% indicated they would continue to use the iFD tool after discharge. The main reasons for not using the iFD tool were short duration of stay, severity of disease and lack of digital literacy.

**Conclusion:**

An implementation of the iFD tool is feasible on principle. Active users gave positive feedback concerning the intervention and most participants claimed to continue using the iFD tool after discharge; however, low usage rates among study participants (42/78, 54%) showed barriers of implementation that have to be addressed and underline the importance of adaptations regarding the use of the intervention in a clinical setting.

Eine Vielzahl internet- und mobilbasierter Interventionen (IMI) wurde speziell für die Bedürfnisse depressiver PatientInnen entwickelt. Die Prüfung der Wirksamkeit und Anwendungssicherheit erleichtert die Auswahl und Verbreitung dieser Tools.

## Hintergrund und Fragestellung

Depressionen sind eine weit verbreitete und schwerwiegende psychische Störung mit einer Lebenszeitprävalenz von ca. 11,6 % [7]. Trotz der hohen Prävalenzraten gelingt nur 50 % der Betroffenen ein Zugang zu geeigneten Behandlungsangeboten, nur ein Drittel erhält am Ende eine depressionsspezifische Behandlung [24].

Eine Möglichkeit zur Verbesserung der Versorgungssituation besteht in der Nutzung von E‑Mental-Health(EMH)-Programmen, welche niedrigschwellig verfügbar, kostengünstig, ortsunabhängig und flexibel in der Anwendung sind [19]. Allerdings werden EMH-Angebote in der Allgemeinbevölkerung generell als weniger hilfreich im Vergleich zu Face-to-face-Therapien wahrgenommen [3], wobei Studien zeigten, dass eine internetbasierte kognitive Verhaltenstherapie (iCBT) ebenso effektiv wie eine Face-to-face-Psychotherapie sein kann [1]. Der positive Effekt von EMH-Programmen verstärkt sich bei therapeutischer Begleitung der Intervention, welche vor allem bei schwergradiger Depression sehr relevant zu sein scheint [14]. Onlineinterventionen eignen sich insbesondere zur Reduktion depressiver Symptome bei leicht- oder mittelgradiger Depression [27], wobei gezeigt werden konnte, dass begleitete iCBT auch bei Personen mit Symptomen einer schweren depressiven Erkrankung zu einer Reduktion der Symptomatik führen kann [22]. Zur Akzeptanzförderung und Erhöhung der Nutzungsbereitschaft eignen sich besonders Programme, welche textbasierte, expertengestützte Informationen integrieren [4]. Im klinischen Behandlungskontext scheint allerdings die Akzeptanz der PatientInnen für EMH-Therapien im Vergleich zu NutzerInnen, welche sich über das Internet informiert und sich eigenständig für die Nutzung entschieden haben, geringer zu sein [26]. Die niedrigere Akzeptanz von EMH-Tools im klinischen Setting wirkte sich in einer Subgruppenanalyse jedoch nicht auf die Effektivität der Intervention aus [15].

In der Routineversorgung ist das Wissen um die Implementierung von Onlineinterventionen bei der Depressionsbehandlung noch gering [10]. Wirksamkeitsnachweise für EMH-Programme liegen für die ambulante Primärversorgung vor [20], wobei eine iCBT in der hausärztlichen Versorgung depressiver Erkrankungen keinen Zusatznutzen erbringt, wenn sie nur durch technischen Support und nicht durch einen Therapeuten begleitet wird [11]. Erste Wirksamkeitsbelege im stationären Kontext lieferte eine Untersuchung zur Kombination eines onlinebasierten Selbstmanagementprogrammes (oSMP) mit einer stationären Psychotherapie [30]. Die Follow-up-Untersuchung ergab bei Fortführung der Intervention im Anschluss an die stationäre Therapie eine höhere Lebensqualität sowie eine geringere depressive Residualsymptomatik [29]. Weiterhin kann iCBT zu einer Erhöhung von Remissionsraten sowie zu einer Reduktion der Wahrscheinlichkeit eines Rezidives beitragen [13]. Allerdings wurden bei Onlineinterventionen hohe Drop-out-Raten beobachtet [31]. Um diese zu reduzieren und die Komplettierungsrate von EMH-Interventionen zu erhöhen, eignet sich insbesondere die Steigerung der präinterventionellen Nutzungsakzeptanz [18].

Ein EMH-Tool ist das kostenfreie Onlineselbstmanagementprogramm „iFightDepression“ (iFD), welches sich aus verschiedenen Workshops konstituiert und auf Techniken der kognitiven Verhaltenstherapie basiert. Das iFD-Tool ist in der ambulanten Versorgung für leichte bis mittelschwere Depression vorgesehen, in dieser Studie wurden aufgrund des stationären Settings PatientInnen mit schwerer Depression inkludiert.

Vor dem Hintergrund der möglichen positiven Aspekte einer Nutzung von EMH-Programmen in der Depressionsbehandlung, aber dem geringen Wissen über ihren Einsatz im stationären Setting, war das Ziel unserer Studie, das iFD-Tool als zusätzliches Behandlungsangebot zur leitliniengerechten depressionsspezifischen stationären Behandlung zu implementieren und zu evaluieren.

## Methodik

Die Studie wurde von Juni 2018 bis Februar 2020 auf der Spezialstation für Affektive Störungen der Klinik und Poliklinik für Psychiatrie und Psychotherapie des Universitätsklinikum Leipzig mit Zustimmung der zuständigen Ethikkommission der Medizinischen Fakultät der Universität Leipzig (AZ: 164/17-ek) durchgeführt.

### Studiendesign

Die Teilnehmenden nutzten das iFD-Tool als Zusatz zum leitliniengerechten Behandlungskonzept der Station mittels eigener mobiler Endgeräte. Die Nutzung sollte in den therapiefreien Zeiten erfolgen. Die Interventionsdauer entsprach der Dauer der stationären Behandlung. Zu Interventionsbeginn (stationäre Aufnahme, T0) füllten die Teilnehmer einen Onlinefragebogen aus, bei Entlassung bearbeiteten die PatientInnen einen Paper-pencil-Fragebogen (T1). Die iFD-Tool-Nutzung war auch nach der Entlassung möglich, eine Evaluation ab diesem Zeitpunkt erfolgte jedoch nicht mehr. Begleitet wurden sie vom Studienverantwortlichen, welcher ein Onlinetraining zu dem iFD-Tool durchlaufen hatte. Die Begleitung erfolgte einmal wöchentlich à 30–60 min im Rahmen einer Gruppentherapie mit Aufklärung und Anleitung zu den Inhalten der Intervention sowie Raum für Fragen und Feedback. Auch das gesamte Pflege- und ÄrztInnen/PsychologInnen-Team durchlief das Onlinetrainingsprogramm zu dem iFD-Tool, damit sie Fragen der StudienteilnehmerInnen jederzeit auch außerhalb des wöchentlichen Meetings beantworten konnten. Nichtteilnehmende konnten freiwillig Ablehnungsgründe angeben.

### Stichprobe

**Figure 1:**
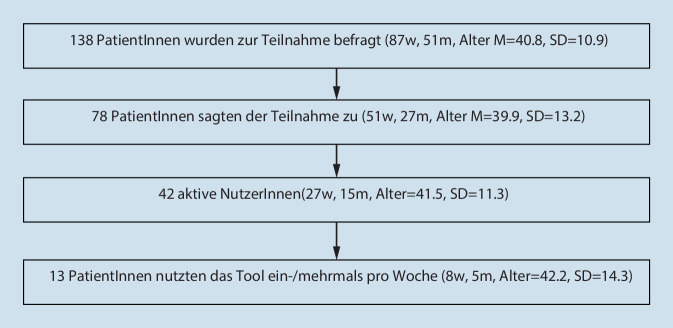

Während der Studiendauer wurden alle neu aufgenommenen und die Einschlusskriterien erfüllenden PatientInnen konsekutiv gefragt. Eingeschlossen wurden 78 PatientInnen (Abb. 1), die unter einer unipolaren Depression unterschiedlichen Schweregrades (ICD-10 F32.0‑3, F33.0-3) oder Dysthymie (F34.1) litten. Die Diagnosestellung erfolgte durch das ärztliche Stationspersonal. Ein- und Ausschlusskriterien sind in Tab. 1 aufgeführt.

**Table 1:** 

Einschlusskriterien	Ausschlusskriterien
Unipolare Depression	Demenz
Dysthymie	Schizophrenie
Alter ≥ 18 Jahre	Suchterkrankung
Längerfristiger stationärer Aufenthalt	Bipolare Störung
Gültige E‑Mail-Adresse	Zwangsstörung
Schriftliche und elektronische Einwilligung	Persönlichkeitsstörung (F60.2 oder F60.3)
	Suizidalität
	Fehlende Medienkompetenz (subjektive Einschätzung/keine eigene E‑Mail-Adresse)

### Das iFD-Tool

Das iFD-Tool ist ein kostenloses, therapeutenbegleitetes oSMP für Erwachsene und Jugendliche basierend auf kognitiver Verhaltenstherapie. Interessierte HausärztInnen, aber auch FachärztInnen für Psychiatrie und Psychotherapie sowie psychologische PsychotherapeutInnen können ein kostenloses Onlinetraining (https://ifightdepression.com/webinar/) durchlaufen und anschließend PatientInnen zur Nutzung freischalten. Das iFD-Tool enthält 6 Kernworkshops und einen Zusatzworkshop (Tab. 2).

**Table 2:** 

Workshop	KVT-basierter Inhalt	Intervention
Denken, Fühlen und Handeln	Verhaltensbeobachtung	Aktivitätenprotokoll
Schlaf und Depression	Schlafverhalten und depressive Symptomatik	Schlaftagebuch
Schöne Dinge planen und unternehmen	Tagesstrukturierung	Planung regelmäßiger Aktivitäten
Dinge erledigen	Problemlösetraining	Antizipieren von Schwierigkeiten und Lösungsmöglichkeiten anstehender Unternehmungen
Negative Gedanken erkennen	ABC-Model	Ereignisse, Gedanken, Reaktionen reflektieren
Negative Gedanken verändern	Kognitive Umstrukturierung	Gedanken infrage stellen
Zusatz: ein gesunder Lebensstil	Psychoedukation: stabilisierende Umgebungsfaktoren	

*KVT* Kognitive Verhaltenstherapie; *ABC*: *A* Activating event, *B* Belief, *C* Consequences

### Untersuchungsinstrumente

Der T0-Fragebogen wurde als Bestandteil des iFD-Tools erhoben. Erfragt wurde die psychiatrische Vorgeschichte und die an den Kontext IMI adaptierte Subskala Allgemeine Behandlungserwartung (BE) des Fragebogens zur Messung der Psychotherapiemotivation (FMP) anhand von 8 Items [25]. Zwei weitere vom iFD-Entwickler-Team konstruierte Items zur Einstellung bezüglich IMI, ein Item zum „shared decision-making“ sowie das Alter und Geschlecht wurden ebenfalls erfasst.

Die Veränderung der depressiven Symptomatik wurde wöchentlich mit dem im iFD-Tool implementierten Patient Health Questionnaire 9 (PHQ‑9, Summenwert: 0–27) erfasst [16].

Der T1-Fragebogen enthielt Fragen zu dem Bearbeitungsumfang, der Art und Zufriedenheit mit der Begleitung, der Nutzungsmodalität, der Interventionszufriedenheit (anhand des ZUF‑8 [23]), der subjektiven Nützlichkeit des iFD-Tools (anhand des USE-Questionnaires [17]) sowie dem Vorhaben der weiteren Nutzung. Nutzungsdaten konnten über das iFD-Tool anonymisiert erfasst werden.

### Statistik

Die statistische Analyse erfolgte deskriptiv mit IBM SPSS Statistics Version 22 (IBM Corp, Armonk, NY, USA). Der PHQ-9-Verlauf wurde mit einer Varianzanalyse bei abhängigen Stichproben analysiert. Die Gruppenunterschiede zum Vergleich der Behandlungserwartung und -zufriedenheit zwischen Frauen und Männern sowie dem Patientenalter < 39 bzw. > 39 Jahren wurden für normalverteilte, varianzhomogene Daten mittels des Zweistichproben-*t*-Tests analysiert, bei Varianzinhomogenität wurde der Welch-Test eingesetzt. Die Zusammenhangsanalyse erfolgte mittels bivariater Korrelationsanalyse. Ein zweiseitiges Signifikanzniveau von α = 0,05 wurde als bedeutsam betrachtet.

## Ergebnisse

### Beschreibung der Stichprobe

Von den 78 Teilnehmenden loggten sich 42 mindestens einmal ein, 36 nutzten das iFD-Tool nicht. Tab. 3 zeigt die demographischen und klinischen Charakteristika der Stichprobe.

**Table 3:** 

Variable	Alle StudienteilnehmerInnen (*n* = 78)	
*Geschlecht *(weiblich), *n* (%)	51 (65,4)	
*Alter *(Jahre), M (SD)	39,9 (13,2)	
	**Aktiv nutzend (*n*** **=** **42)**	**Nicht nutzend (*n*** **=** **36)**
*Geschlecht* (weiblich), *n* (%)	27 (64,3)	24 (66,7)
*Alter *(Jahre), M (SD)	41,5 (11,3)	38,2 (12,0)
*Depressive Episode in der Vergangenheit n* (%)	26 (61,9)	
*Therapieerfahrung*		
Pharmakotherapie, *n* (%)	37 (88,1)	
Psychotherapie, *n* (%)	35 (83,3)	
Andere^a^, *n* (%)	12 (28,6)	
*Median selbstberichteter depressiver Episoden in der Vergangenheit, n* (SW)	3,0 (1–8)	

*M* Mittelwert*, SD* Standardabweichung*, SW* Spannweite

^a^Andere Therapieerfahrungen: Elektrokrampftherapie, Schlafentzug, ambulante Ergotherapie, ambulante psychiatrische Pflege

### Pretreatment

Daten zur Interventionserwartung liegen von 42 Personen vor, Tab. 4 zeigt die Auswertung der Einzelitems. Unterschiede zwischen aktiven und inaktiven PatientInnen konnten bezüglich Alter und Geschlecht nicht festgestellt werden. Die häufigsten Gründe für Inaktivität trotz Studienteilnahme waren fehlende Zeit (33 %, 12/36), der Schweregrad der Depression (19 %, 7/36) und die Länge der Textbausteine (14 %, 5/36). Da der T0-Fragebogen nur bei Bearbeitung des iFD-Tools erfasst werden konnte, liegen zu den inaktiven NutzerInnen keine Daten zur Therapievorerfahrung vor. Die Zustimmung wurde mit einer 5‑stufigen Likert-Skala erfasst: 1 „strongly agree“, 2 „agree“, 3 „neutral“, 4 „disagree“, 5 „completely disagree“. Die Items 1, 2, 4, 6, 7, 8, 9, 10, 11 wurden von 1–5 bewertet, die Items 3 und 5 wurden invers skaliert. Die allgemeine Behandlungserwartung lag bei *M* = 26,23 (*SD* = 3,25). Es wurde angenommen, dass es zwischen Geschlecht und Alter keine Differenzen bezüglich Erwartung und Zufriedenheit gibt. Im *T*-Test (Tab. 7) unterschieden sich weder Frauen/Männer (t_[39]_ = −0,77, *p* = 0,45) noch Patienten unter/über 39 Jahren (t_[38]_ = 1,17, *p* = 0,25) signifikant.

**Table 4:** 

Item	Median/MW (SD)
*Behandlungserwartung*	
1 Mal ausspannen hilfreicher als oSMP	3/3,3 (0,98)
2 Nur medizinische Behandlung kann meine Beschwerden mindern	2/2,5 (1,1)
3 Kann noch vieles lernen, was hilft, meine Krankheit zu bewältigen	4,5/4,2 (0,91)
4 Lösung meiner Probleme hilfreicher als oSMP	3/2,8 (1,0)
5 Kann aktiv zur Beschwerdebesserung beitragen	4/4,2 (0,79)
6 Arzt wäre passender als oSMP	2/2,4 (0,74)
7 Glaube kaum, dass oSMP hilft	4/3,7 (0,81)
8 Medikament/Operation wären mir lieber als oSMP	3/3,2 (1,1)
*Einstellung gegenüber IMI*	
9 Sehe Vorteile in der Nutzung von oSMP	2/2,1 (0,73)
10 Persönlicher Behandlerkontakt ist wichtig	1/1,5 (0,84)
*Entscheidungsprozess*	
11 War an der Nutzungsentscheidung aktiv beteiligt	1/1,6 (0,91)

*oSMP* onlinebasiertes Selbstmanagementprogramm, *MW* Mittelwert, *SD* Standardabweichung, *IMI* internet- und mobilbasierte Intervention

Der initiale PHQ-9-Wert zeigte keinen signifikanten Zusammenhang mit der Behandlungserwartung (r = −0,19; *p* = 0,29).

### Verlauf der Depressionsschwere

**Figure 2:**
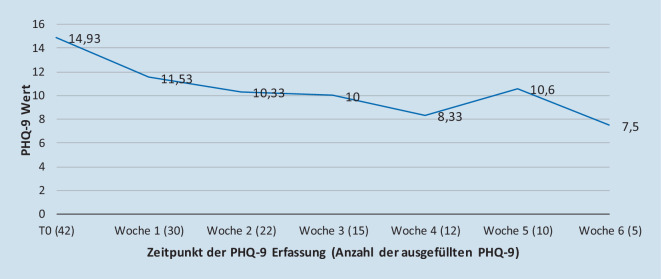

Der Anteil schwergradig Erkrankter war mit 54,8 % verglichen mit der Gesamtkohorte hoch (Tab. 5). Im Mittel lag der initiale PHQ‑9 Wert bei *M* = 14,93 (*SD* = 5,11) und fiel während des stationären Aufenthaltes von 14,93 auf 7,5 (−7,43; 95 %-Konfidenzintervall −8,14 bis −6,73; *p* < 0,05). Die Anzahl der ausgefüllten Bögen pro Woche nahm mit der Zeit ab, was eine abnehmende Nutzerrate im zeitlichen Verlauf impliziert, da der PHQ‑9 wöchentlich obligat abgefragt wurde (Abb. 2).

**Table 5:** 

Minimal *n* (%)	1 (2,3)
Mild *n* (%)	5 (11,9)
Mittelgradig *n* (%)	13 (31,0)
Schwer *n* (%)	23 (54,8)

^a^Der Summenwert des PHQ‑9 lässt sich in 4 Kategorien zur Abbildung der Stärke der depressiven Symptomatik einteilen: 0–4 = minimal; 5–9 = mild; 10–14 = mittelgradig; 15–27 = schwer

### Posttreatment

Drei Teilnehmende hatten die Nutzung abgeschlossen, 21 hatten dies noch vor und 18 hatten die Nutzung unterbrochen, um sie bei Bedarf fortzusetzen. 29 PatientInnen hatten das iFD-Tool seltener als einmal pro Woche genutzt, 6 PatientInnen nutzten das iFD-Tool einmal pro Woche, 4 der Teilnehmenden gaben an, das iFD-Tool mehrmals wöchentlich genutzt zu haben. 2 PatientInnen nutzten das iFD-Tool täglich und eine Person mehrmals täglich. Im Median wurde das iFD-Tool für 10 min und 3 Sessions genutzt. Die Interventionszufriedenheit lag bei *M* = 23,1 (*SD* = 4,76, Summenwerte: 8–32). Tab. 6 zeigt die Auswertung der Einzelitems, diese wurden 4‑stufig (1: geringe Merkmalsausprägung bis 4: hohe Merkmalsausprägung) erfasst und sind in prozentualer Zustimmung angegeben.

**Table 6:** 

Item	Zustimmung (%)
Wie beurteilen Sie die Qualität von iFD?	69,9
Haben Sie mit iFD die Art von Unterstützung erhalten, die Sie wollten?	61,4
Hat iFD Ihren Bedürfnissen entsprochen?	58,1
Würden Sie das iFD-Tool empfehlen?	63,7
Wie zufrieden sind Sie mit dem Ausmaß der Hilfe?	60,4
Hat das Tool geholfen, angemessener mit Problemen umzugehen?	60,4
Wie zufrieden sind Sie mit dem Tool?	64,3
Würden Sie das Tool wieder benutzen?	60,4

*iFD* „iFightDepression“

Gering fiel die Bewertung der subjektiven Nützlichkeit aus (*M* = 15,35, *SD* = 5,96, Summenwerte: 7–35), ebenso wie die 2 Items der Skala „usefulness“ (*M* = 6,61, *SD* = 2,44, Summenwerte: 2–14). 28 Teilnehmende gaben an, das iFD-Tool poststationär weiter nutzen zu wollen. Hauptgründe gegen die Fortführung waren geringe Medienaffinität, fehlendes Therapeutenfeedback, ausbleibender Therapieeffekt und hoher Zeitaufwand.

### Behandlungszufriedenheit

Zwischen den Geschlechtern (t_[28]_ = 0,94, *p* = 0,35) und dem Alter (t_[28]_ = −0,31, *p* = 0,76) gab es keinen signifikanten Unterschied der Behandlungszufriedenheit in Bezug auf das iFD-Tool (Tab. 7).

**Table 7:** 

	Behandlungserwartung M (SD)	Behandlungszufriedenheit M (SD)
Frauen	26,0 (3,16)	22,2 (3,20)
Männer	26,8 (3,53)	23,4 (3,10)
Alter > 39 Jahre	26,9 (2,26)	22,8 (3,26)
Alter < 39 Jahre	25,8 (3,83)	22,4 (3,18)

*SD* Standardabweichung, *M* Median

## Diskussion

Das Ziel unserer Studie war, den Nutzen und die Anwendbarkeit des iFD-Tools für depressive PatientInnen im stationären Setting zu untersuchen. 62 % (48/78) der Studienteilnehmenden registrierten sich zur Nutzung des iFD-Tools. Insgesamt nutzten 54 % (42/78) der TeilnehmerInnen das Angebot mindestens einmal während ihres Aufenthaltes, aber nur 17 % (13/78) der PatientInnen nutzten das iFD-Tool ein- oder mehrmals pro Woche. Ähnlich geringe Nutzungsraten zeigen andere Studien zu IMI im stationären [9, 31] und ambulanten Setting [20]. Verglichen mit dem notwendigen Aufwand für die Toolimplementierung, welche Personalschulungen und eine regelmäßige Betreuung der NutzerInnen erforderte, ist die Nutzungsrate im stationären Setting aktuell als eher gering zu bewerten. Im Rahmen zunehmender Digitalisierungsprozesse und der pandemiebedingten Einschränkungen psychiatrischer Behandlungsangebote bleibt abzuwarten, ob IMI in Zukunft eine erhöhte Nachfrage und Nutzungsbereitschaft, auch im stationären Sektor, erfährt.

### Für wen ist das iFD-Tool geeignet?

Barrieren für die Nutzung waren eine kurze Dauer des stationären Aufenthaltes, fehlende subjektive Medienkompetenz und eine schwere depressive Symptomatik. Als Hauptgründe für die Nichtnutzung des iFD-Tools trotz Studienteilnahme wurden Zeitmangel aufgrund anderer Face-to-face-Therapieangebote, der Schweregrad der Depression und die Länge der Texte seitens der Patienten angegeben. Als akzeptanz- und compliancefördernd hat sich die Demonstration des iFD-Tools und der technische Support durch das Studienteam im Rahmen einer eigens hierfür etablierten Gruppentherapie erwiesen. Diese Erfahrung deckt sich mit der besseren Wirksamkeit von begleiteten IMI [14] und der Steigerung der Akzeptanz durch den Erhalt expertengestützter Informationen und Empfehlungen [4].

Die Werte des PHQ‑9 nahmen im Verlauf der stationären Therapie sukzessive ab. Aufgrund der multimodalen Behandlung und dem abnehmenden Fragebogenrücklauf lässt sich dies jedoch nicht unbedingt alleinig auf das iFD-Tool zurückführen.

Mit 26,23 von 40 Punkten fiel der Erwartungswert leicht überdurchschnittlich aus, ähnlich zu Untersuchungen von IMI im ambulanten Setting [6, 8]. Die Teilnehmenden signalisierten im Pretreatment-Fragebogen Lern- und Handlungsbereitschaft bezüglich der Intervention.

### Aspekte der Nutzererfahrung im stationären Setting

Die PatientInnen präferierten eine Face-to-face-Therapie, was im Rahmen des klinischen Settings nachvollziehbar ist. Die PatientInnen sahen sich als aktiv entscheidend im Nutzungsprozess an, erkennbar an der hohen Zustimmung zur Einnahme einer aktiven Rolle im Entscheidungsprozess (Tab. 4, Item 11), was für eine Fortführung der Nutzung relevant ist und in einer Untersuchung der IMI „color your life“ [8] ähnlich evaluiert wurde.

Die moderat positive Interventionszufriedenheit deckt sich mit den Angaben zur iFD-Tool-Nutzung im ambulanten Setting [21]. Im stationären Rahmen erzielte „moodgym“ eine ähnliche Nutzerzufriedenheit [9], ebenso andere EMH-Interventionen [2, 12]. Möglicherweise steigen die Zufriedenheitswerte, wenn die Inhalte solcher Tools mit den weiteren stationären Therapieangeboten besser abgestimmt werden [5], was sich im Rahmen des „blended-treatment“, also der Kombination aus Face-to-face-Psychotherapie und einer Onlineintervention, als wirksam erwiesen hat [30].

Eine geringe Komplettierungsrate der Onlineintervention findet sich ebenso in anderen EMH-Bereichen [28]. Dies könnte generell ein Hinweis für die Notwendigkeit einer bedarfsgerechten Anpassung solcher Tools sein.

Die Nützlichkeit wurde als gering beurteilt, wohingegen die Auswertung jedes Workshops einzeln ergab, dass 38 Personen einen oder mehrere Workshops als hilfreich ansahen, während nur 4 Teilnehmer einen oder mehrere Workshops als nicht hilfreich oder gar belastend empfanden.

Trotz der moderat bewerteten Gesamtnützlichkeit gaben 67 % der aktiven Nutzer an, das iFD-Tool poststationär weiter nutzen zu wollen, was aufgrund der fehlenden Nachverfolgung nicht überprüft werden konnte. Die Möglichkeit der langfristigen Nutzung ist ein relevanter Aspekt und kann zur nachhaltigen Symptomreduktion beitragen [29], sollte aber in weiteren Studien untersucht werden.

Limitationen der Ergebnisse dieser Studie sind die geringen Fallzahlen und die fehlende Kontrolle möglicher Medikamenten- und Psychotherapieeinflüsse. Des Weiteren ergaben sich aufgrund des naturalistischen Studiendesigns unterschiedlich lange Anwendungszeiten, welche die Behandlungsrealität eines stationären Settings abbilden, allerdings auch die Vergleichbarkeit erschweren.

Die Limitationen ergeben sich teilweise aufgrund der Wahl eines naturalistischen Settings, welches eine praxisnahe Evaluierung des Tools im klinischen Alltag ermöglichte. Die Anwendung auch bei schwerer depressiver Symptomatik führte einerseits zu einer symptombedingten Einschränkung der Anwendbarkeit seitens der Nutzer, ermöglichte aber andererseits auch erste Erfahrungen mit einer Nutzung des iFD-Tools bei schwerer depressiver Episode.

## Fazit für die Praxis

- Die Implementierung des iFD-Tools bei depressiver Erkrankung erwies sich im stationären Rahmen als machbar und führte bei Nutzung zu vorwiegend positivem Feedback seitens der PatientInnen, ergab jedoch insgesamt geringe Nutzungsraten.
- Barrieren für die Anwendung im stationären Setting waren eine kurze Dauer des stationären Aufenthalts, Zeitmangel aufgrund anderer Face-to-face-Therapieangebote, eine besonders schwere depressive Symptomatik und eine fehlende Medienkompetenz der PatientInnen.
- Eine gezielte Ansprache medienaffiner Personen erhöht die Wahrscheinlichkeit einer Nutzung des iFD-Tools. Um die Compliance zu erhöhen, sollte die Intervention therapeutisch begleitet werden. Dies kann im stationären Setting auch als Gruppenformat erfolgen.
- Eine poststationäre Fortführung der Nutzung wurde von den meisten aktiven Nutzern gewünscht. Dies kann im Entlassungsmanagement integriert werden und sollte im Rahmen zukünftiger Studien untersucht werden.
